# Association between childhood maltreatment and obsessive-compulsive disorder comorbid with eating disorders: a cross-sectional study

**DOI:** 10.1186/s40337-024-01090-0

**Published:** 2024-09-06

**Authors:** Salma Attar, Jinane Jomaah, Rhéa El Khoury, Colin Cordahi, Maude Seneque, Philippe Courtet, Rami Bou Khalil, Sebastien Guillaume

**Affiliations:** 1 Faculty of Pharmacy, Saint-Joseph University, Beirut, Lebanon; 2 Department of Psychiatry, Faculty of Medicine, Saint Joseph University, Achrafieh, Beirut, Lebanon; 3 Department of Psychiatry, Hotel-Dieu de France Hospital, Achrafieh, P.O. box: 166830, Beirut Lebanon; 4 Institute of Functional Genomics, University of Montpellier, CNRS, INSERM, Montpellier, France; 5 Department of Psychiatric Emergency and Acute Care, Lapeyronie Hospital, CHRU, Montpellier, France

**Keywords:** Obsessive-compulsive disorder, Eating disorder, Comorbidity, Trauma, Childhood traumatic experiences, Sexual abuse

## Abstract

**Background:**

Obsessive-compulsive disorder (OCD) and eating disorders (ED) share common features, including the presence of obsessions and compulsions, and they often co-occur. Additionally, there is a significant comorbidity between ED and childhood traumatic experiences (CTE), as well as between CTE and OCD. Various biological and environmental factors have been proposed to explain the connection between ED, OCD, and CTE. This study explores the link between CTE and the comorbidity of ED and OCD, with the hypothesis that specific types of CTE may increase the risk of developing OCD in individuals with ED.

**Methods:**

Participants (*N* = 562) were enrolled at an eating disorder unit in Montpellier, France, between March 2013 and January 2020. The Childhood Trauma Questionnaire (CTQ), Eating Disorder Examination Questionnaire (EDE-Q), and Mini International Neuropsychiatric Interview (MINI) were used to evaluate childhood maltreatment, assess clinical characteristics associated with ED, and categorize participants into two groups: patients with and without OCD.

**Results:**

Bivariate analysis revealed that patients with comorbid ED and OCD had higher EDE-Q scores (*p* < 0.001), more anxiety disorders (*p* < 0.001), depressive disorders (*p* = 0.02), post-traumatic stress disorder (PTSD) (*p* < 0.001), and a higher incidence of sexual abuse (*p* < 0.001) and physical neglect (*p* = 0.04) compared to those without OCD. Multivariate analysis showed that the association between CTE and OCD was influenced by the presence of an anxiety disorder (*p* = 0.01) and a higher EDE-Q total score (*p* = 0.03), with a significant association with a history of sexual abuse (*p* = 0.04).

**Conclusions:**

This demonstrates that CTE increases the risk of comorbid OCD in ED patients, correlating with more clinically severe ED and a higher likelihood of anxiety disorders.

## Abstract (plain language summary)

This study looked at the link between childhood trauma and the occurrence of both obsessive-compulsive disorder (OCD) and eating disorders (ED). We studied 562 participants who were being treated for eating disorders in Montpellier, France, over a seven-year period. We used various questionnaires to evaluate their childhood experiences, eating disorder symptoms, and the presence of OCD. We found that people with comorbid OCD and ED had more severe eating disorder symptoms and higher rates of anxiety, depression, and PTSD compared to those with just an eating disorder. These individuals were also more likely to have experienced sexual abuse and physical neglect during childhood. Our analysis showed that the connection between childhood trauma and OCD in patients with eating disorders was influenced by the presence of anxiety disorders and more severe eating disorder symptoms. Specifically, a history of sexual abuse was a significant factor. In summary, childhood trauma, especially sexual abuse, increases the risk of having both OCD and an eating disorder. This highlights the importance of screening for OCD and addressing past traumas in patients with eating disorders to provide better, more personalized care.

## Background

Obsessive-compulsive disorder (OCD) is a chronic mental disorder characterized by recurring, intrusive, contradictory thoughts (obsessions) and repetitive, ritualistic behaviors (compulsions) aimed at reducing the anxiety and depression caused by these thoughts [1, 2]. Similarly, eating disorders (ED) are serious mental disorders that impair cognitive function, judgment, emotional stability, and daily living activities [3, 4]. Some ED behaviors and rituals can be considered compulsive, often performed to alleviate anxiety and distress driven by obsessive thoughts related to food, diet, body weight, and shape [5]. An additional diagnosis of OCD in ED patients is considered only if obsessions and compulsions are unrelated to food or weight [6]. The scientific literature indicates a high comorbidity between OCD and ED. A recent meta-analysis disclosed an aggregate lifetime OCD prevalence of 13.9% and a current OCD prevalence of 8.7% among ED patients. ED patients are 8.9 times more likely to develop lifetime OCD traits, with a greater risk observed in anorexia nervosa (AN) patients [7].

Childhood traumatic experiences (CTE), including emotional, physical, and sexual abuse, neglect, witnessing domestic violence, and life-threatening accidents, are defined as exposure to actual or threatened death, serious injury, or sexual abuse according to the Diagnostic and Statistical Manual of Mental Disorders (DSM-IV and DSM-5 TR) [6, 8, 9]. Studies have shown an association between ED and CTE, suggesting that the subtype, pattern, age of occurrence, and intensity of trauma can influence the development of ED [3, 5, 10]. CTE can cause both biological and relational vulnerabilities, potentially leading to specific psychopathological imbalances. These imbalances may result in a unique subtype of ED, characterized by distinct psychological and biological features and a specific developmental course. Patients with ED and a history of CTE have also been found to exhibit increased obsessive and compulsive symptoms [11]. Higher levels of CTE are also reported in subjects with OCD compared to healthy controls [12–14]. Several studies have documented a strong relationship between CTE and OCD severity [15–17].

Researchers have explored the potential mediating roles of shared neurobiological, genetic, and psychosocial vulnerabilities to better understand the relationships between CTE, ED, and OCD. These mechanisms likely interact, with one example being the dysregulation of the limbic-hypothalamic-pituitary-adrenal (LHPA) axis after trauma exposure. This dysregulation leads to the release of corticotrophin-releasing hormone (CRH), which indirectly stimulates the locus coeruleus-noradrenergic (LC-NA) system via the central amygdala [18, 19]. Functional alterations of the NA system have been consistently observed in patients with compulsive disorders [18, 20], and HPA axis dysregulation has been widely described in ED, often linked to specific ecophenotype subgroups [21]. On the other hand, understanding the gene-environment interplay and highlighting the impact of CTE on personality characteristics and psychosocial factors as vulnerability traits for ED and OCD is essential. Vulnerability factors for ED include dissociation [8], emotional dysregulation [22], self-criticism, dysfunctional attitudes and behaviors [23, 24], and body dissatisfaction [5]. For OCD, vulnerability traits such as neuroticism, meticulousness, introversion, and anxiety symptoms are significant [14]. These findings align with the hypothesis that CTE triggers intrinsic alterations tied to the traumatic experience itself, influencing the development of psychopathology regardless of the specific disorder that manifests [11].

Our primary hypothesis is that the presence of CTE in patients with ED is associated with the development of comorbid OCD, with specific subtypes of CTE being more implicated in this association. Our secondary hypothesis posits that more clinically severe ED is more frequently associated with the comorbidity of ED and OCD in patients with a history of CTE.

## Materials and methods

### Participants

Patients diagnosed with ED according to DSM-5 criteria were selected from an ED unit between March 2013 and January 2020 in Montpellier, France. All eligible patients received information about the study and provided consent, either directly or through signed parental consent for minors. The inclusion criteria included ages 15 to 70 years, French-speaking, and a confirmed ED diagnosis according to DSM-5, where ED are characterized by an excessive focus on body shape and weight for self-esteem, and involve unhealthy eating behaviors like fasting, binge eating, and urging [25]. Patient eligibility was confirmed through diagnostic review, with patients either presenting with continuous restriction of energy intake, a fear of gaining weight or becoming fat, and a distorted perception of one’s own weight or body shape ; or presenting with frequent episodes of binge eating, regular use of inappropriate compensatory behaviors to avoid weight gain, and an excessive focus on body shape and weight [25]. All consecutive patients, without restrictions regarding type, chronicity, severity of their ED, or sociodemographic parameters, were admitted and assessed in the daycare unit in order to tailor a management plan by the specialized team at the university hospital in Montpellier, France. Exclusion criteria included refusal to participate, intellectual or psychotic disorders, alcohol abuse, and severe physical comorbidities (e.g., autoimmune diseases, chronic infections affecting white blood cell counts). The database used for this study originated from two large groups of studies approved by the ethics committee South Mediterranean IV (reference number 11-04-SC) and CPP South East VI (reference number AU13-13). The two groups were recruited using the same inclusion and exclusion criteria, with the first group between 2013 and 2017 and the second group between 2017 and 2020. The studies were conducted in accordance with the Declaration of Helsinki.

### Measures

A multidisciplinary clinical examination was conducted by a professional mental health unit in one day. The diagnosis of ED was established through an unstructured clinical examination by psychologists, psychiatrists, and nutritionists, as well as a structured assessment using the Mini International Neuropsychiatric Interview (MINI, version 5.0.0). Participants completed two questionnaires: The Eating Disorder Examination Questionnaire (EDE-Q) and the Childhood Trauma Questionnaire (CTQ).

The MINI 5.0.0 is a brief, structured diagnostic interview designed to assess the major Axis I psychiatric disorders in DSM-IV and ICD-10. At the time of the recruitment, this version of the MINI was available. It provides a reliable and valid method for diagnosing mental health disorders [26].

The EDE-Q is a 28-item self-report questionnaire on the psychopathology of eating disorders, assessing disordered eating attitudes and behaviors over the past 28 days. It consists of four subscales: restraint (5 items), eating concern (5 items), shape concern (8 items), and weight concern (5 items). An overall score is calculated from the average. A cut-off score of ≥ 4 indicates clinical significance, meaning that patients with an EDE-Q score of four or higher were considered to have clinically severe ED [27]. Research has demonstrated the reliability and validity of the EDE-Q scores in assessing ED symptoms and distinguishing between individuals with and without ED [28].

The CTQ consists of 28 items for the retrospective diagnosis of childhood trauma, comprising five subscales to detect different types of trauma (physical abuse, sexual abuse, emotional abuse, physical neglect, and emotional neglect). It has demonstrated strong psychometric proprieties in clinical samples [29]. Each category is scored using a 5-point Likert scale from 1 (never true) to 5 (very often true). Higher scores indicate severer childhood trauma.

The internal consistency of the questionnaires was assessed with Cronbach’s alpha. The EDE-Q had an acceptable alpha of 0.716. The CTQ total scale and its subscales generally demonstrated good to excellent reliability: emotional abuse (α = 0.827), physical abuse (α = 0.862), sexual abuse (α = 0.957), and emotional neglect (α = 0.911). However, the physical neglect subscale had a low alpha of 0.436.

According to the MINI, participants were divided into two groups. Those with [OCD (+)] exhibited at least one of the following criteria: presence of obsessions (recurrent and persistent thoughts, impulses, or images) and/or compulsions (repetitive behaviors or mental acts they felt driven to perform) that were time-consuming (at least one hour per day), resulting in distress and interference with the patient’s normal routine. We targeted individuals with current OCD. Furthermore, the obsessive-compulsive symptoms should not be secondary to an ED. Conversely, those classified as [OCD (-)] showed no characteristic signs.

### Study design

This study employed a cross-sectional design, analyzing data collected from patients diagnosed with ED between March 2013 and January 2020 at an ED unit in Montpellier, France. The design facilitated the assessment and comparison of several sociodemographic, clinical and childhood trauma parameters in ED patients while dividing them between those with OCD [group OCD(+)] and those without OCD [group OCD(-)]. A multivariate analysis was conducted in order to assess, after controlling for potential confounding factors, what characteristics are more associated to the comorbidity of ED and OCD.

### Statistical analysis

Quantitative variables that significantly departed from normality assumptions (assessed with the Shapiro-Wilk test and quartile–quartile plots) were expressed as medians with interquartile ranges (IQR: Q1–Q3). We compared the distribution of key variables across the four ED groups: AN, bulimia nervosa (BN), binge-eating disorder (BED), and other ED; a one-way ANOVA test was used for continuous variables, and a chi-square test was used for categorical variables. All continuous variables were not normally distributed. In this study, OCD (+/-) was considered the dependent variable. We did an effect size evaluation, using Cohen’s d for continuous variables and Odds Ratios (OR) for categorical variables, to compare the two groups. Each independent variable was analyzed alone in a logistic regression model using the Logit function. We also used the Akaike Information Criterion (AIC) to measure the quality of the statistical models. The AIC penalizes models according to the number of parameters to satisfy the criterion of parsimony. The model with the lowest AIC was considered the most parsimonious, balancing quality of fit and model complexity to limit over-fitting effects.

In the multivariate analysis, to differentiate between the different subtypes of CTE, the CTQ was considered a continuous variable, and its effect was evaluated accordingly. To determine whether the association between CTE and OCD was accounted for by an overlap with anxiety symptoms, depressive disorder, PTSD, and ED, we examined the association between those explanatory variables (selected based on *p* < 0.15) and three subtypes of childhood trauma from the previous bivariate analysis results (emotional abuse, sexual abuse, and physical neglect). We evaluated the five types of childhood trauma; however, the three subtypes of childhood trauma retained for the multivariate analysis were those that showed significant differences between the two groups or had a p-value < 0.15, indicating a trend. We also examined the association between these variables and the total CTQ score, as well as the sum of the three subtypes of childhood trauma. In cases of overlap between different subtypes of CTE, the lowest AIC indicated which subtype was more associated with the comorbidity of ED and OCD. AIC was calculated to compare the relative quality of all models proposed in the study using the formula: AIC = n x ln(SSres) + 2 K. All statistical analyses were performed using JASP 0.16.4.0 and SPSS 28.0 software.

## Results

### Sample study

A total of 600 participants with ED were consecutively included in the study (Table 1). Thirty-eight of these participants were excluded due to the lack of available OCD screening, resulting in a final sample size of 562. Three hundred and four participants (54.1%) were diagnosed with AN, 135 participants (24.1%) were diagnosed with BN, and 57 participants (10.1%) had a BED. The remaining 66 participants (11.7%) were classified as having ‘other ED’. Our findings revealed significant differences in several parameters, such as age (*p* < 0.001), current BMI (*p* < 0.001), resting energy expenditure (*p* < 0.001), percentage of fat mass (*p* < 0.001), current substance use disorder (*p* = 0.001), and the total CTQ score (*p* = 0.004). Based on the MINI results, 73 participants were classified in the [OCD (+)] group, while 489 were classified in the [OCD (-)] group. The mean score on the CTQ was 44.61 (low to moderate), with a minimum of 25 and a maximum of 125. In the sample as a whole, the most commonly reported subtype of childhood trauma was emotional neglect (12 [8–16]), followed by emotional abuse (9 [6–14]). Physical neglect (7 [5–9]), sexual abuse (5 [5, 6]), and physical abuse (5 [5–7]) were the least endorsed.

### Bivariate analysis

In the bivariate analysis, we compared the [OCD (+)] (*N* = 73) and [OCD (-)] (*N* = 489) groups (Table 2). Four variables were statistically significant: the EDE-Q total score (*p* < 0.001), depressive disorder at the time of investigation (*p* = 0.02), anxiety disorder at the time of investigation (*p* < 0.001), and PTSD at the time of investigation (*p* < 0.001).

**Table 1:** Demographic and clinical characteristics of the whole sample and comparison between ED subtypesª

Variable	*N* = 562	AN = 304 (54.1%)	BN = 135 (24.1%)	BED = 57 (10.1%)	Other ED = 66 (11.7%)	Statistics^b^	*P*-value
Age (Median, ITQ)	24 [20–34]	23 [19–31]	27 [21–36]	30 [24–43]	23 [20–34]	9.03	< 0.001*
Sex Men Women	33(5.9%) 529(94.1%)	17(5.6%) 287(94.4%)	7(5.2%) 128(94.8%)	3(5.3%) 54(94.7%)	6(9.1%) 60(90.9%)	1.43	0.69
Current BMI (kg/m^2^) (Mean)	20.39	17.12	22.38	32.94	20.5	279.34	< 0.001*
Resting energy expenditure (kcal) (Mean)	1256.48	1131.99	1347.15	1631.19	1303.55	88.21	< 0.001*
Percentage of fat massy (Mean)	26.31	21.06	30.09	42.4	27.05	103.22	< 0.001*
Eating disorder examination-questionnaire (EDE-Q) total score (Mean)	3.33	3.09	4.13	3.78	2.38	24.92	< 0.001*
Current depressive disorder	163 (29%)	91 (29.9%)	35 (25.93%)	20 (35.09%)	17 (25.76%)	1.94	0.58
Current anxiety disorder	259 (46.08%)	137 (45.07%)	62 (45.93%)	30 (52.63%)	30 (45.45%)	1.21	0.77
Current post-traumatic stress disorder	44 (7.83%)	21 (6.91%)	10 (7.41%)	4 (7.02%)	9 (13.64%)	4.26	0.23
Current obsessive-compulsive disorder	73 (12.98%)	39 (12.8%)	22 (16.29%)	6 (10.53%)	6 (9.09%)	2.51	0.47
Current substance use disorder	42 (7.47%)	19 (6.25%)	20 (14.8%)	2 (3.51%)	1 (1.51%)	16.55	0.001*
Current nicotine consumption	210 (37.36%)	121 (39.8%)	54 (40%)	19 (33.33%)	22 (33.33%)	4.83	0.57
Personal history of suicide attempts	137 (24.37%)	59 (19.4%)	41 (30.37%)	15 (26.32%)	22 (33.33%)	10.49	0.015*
Emotional abuse (Median, ITQ)	9 [6–14]	8 [6-12.75]	11 [7–13]	11.5 [7–16]	8.5 [6–13]	6.67	< 0.001*
Physical abuse (Median, ITQ)	5 [5–7]	5 [5–6]	5 [5-7.5]	5 [5-6.5]	5 [5–6]	1.25	0.29
Sexual abuse (Median, ITQ)	5 [5–6]	5 [5–6]	5 [5–7]	5 [5-8.5]	5 [5–5]	0.86	0.46
Emotional neglect (Median, ITQ)	12 [8–16]	10 [7.5–15]	14 [10–17]	13 [11–16]	12 [8.5–17]	5.73	< 0.001*
Physical neglect (Median, ITQ)	7 [5–9]	7 [5–9]	8 [6–10]	7.5 [6–11]	7 [6–9]	3.19	0.024*
Total CTQ score (Median, ITQ)	40 [33–51]	38 [31–50]	45 [37–55]	45 [37–53]	39 [33-49.5]	4.56	0.004*

ªData are given as number of each group. Mean was used for the data with a symmetric distribution and median was used for skewed data

^b^A one-way ANOVA test was used for continuous variables, and a chi-square test was used for categorical variables

*Asterisks indicate statistical significance (*p*<0.05)

*Abbreviations* AN = Anorexia nervosa ; BN = Bulimia Nervosa ; BED = Binge-eating disorder BMI = Body Mass Index ; ED = eating disorders ; ITQ = Interquartile

Two subscales of direct trauma exposure, physical neglect (*p* = 0.04) and sexual abuse (*p* < 0.001), were also associated with the [OCD (+)] group. However, physical and emotional abuse, as well as emotional neglect, were not associated with the [OCD (+)] group.

Overall, patients with comorbid ED and OCD, compared to those without OCD, tend to have more clinically severe ED (EDE-Q total score: 4.68 in [OCD (+)] vs. 3.52 in [OCD (-)]; *p* < 0.001), more anxiety disorders (68.49% in [OCD (+)] vs. 42.74% in [OCD (-)]; *p* < 0.001), a higher prevalence of depressive disorder at the time of investigation (41.10% in [OCD (+)] vs. 27.20% in [OCD (-)]; *p* = 0.02), and PTSD at the time of investigation (20.55% in [OCD (+)] vs. 5.93% in [OCD (-)]; *p* < 0.001), as well as the aforementioned history of sexual abuse and physical neglect.

**Table 2:** Bivariate analysis comparing ED patients’ demographic and clinical variables between [OCD (+)] and [OCD (-)] groups

Variable	OCD (+) *N* = 73	OCD (-) *N* = 489	*P*-value
Age (Mean)	29.03	28.20	0.55
Sex Men Women	3 (4.1%) 70 (95.89%)	30 (6.13%) 459 (93.86%)	0.49
Current BMI (kg/m^2^) (Mean)	20.19	20.42	0.76
Resting energy expenditure (kcal) (Mean)	1247.41	1257.85	0.77
Percentage of fat massy (Mean)	26.91	26.23	0.63
Eating disorder examination-questionnaire (EDE-Q) total score, (Mean, ITQ)	4.68 [3.30–5.26]	3.52 [1.97–4.44]	< 0.001*
Current depressive disorder	30 (41.1%)	133 (27.2%)	0.02*
Current anxiety disorder	50 (68.49%)	209 (42.74%)	< 0.001*
Current post-traumatic stress disorder	15 (20.54%)	29 (5.93%)	< 0.001*
Current substance use disorder	8 (10.95%)	34 (6.95%)	0.20
Current nicotine consumption	32 (43.83%)	178 (36.4%)	0.19
Personal history of suicide attempts	22 (30.13%)	115 (23.51%)	0.19
Emotional abuse (Mean)	11.65	10.32	0.07
Physical abuse (Mean)	7.18	6.71	0.38
Sexual abuse (Median, ITQ)	7 [5-14.25]	5 [5–6]	< 0.001*
Emotional neglect (Mean)	13.07	12.32	0.29
Physical neglect (Median, ITQ)	8 [6-10.50]	7 [5–9]	0.04*

*Asterisks indicate statistical significance (*p*<0.05)

*Abbreviations* ED = Eating Disorder; OCD = Obsessive Compulsive Disorder; OCD (+) = comorbid OCD; OCD (-) = non-comorbid OCD; BMI = Body Mass Index; ITQ = Interquartile

The results show that for the continuous variables, the effect sizes wew generally small, with Cohen’s d values of 0.125 for age, 0.125 for current BMI, 0.127 for resting energy expenditure, 0.137 for EDE-Q total score, and 0.127 for the percentage of fatty mass. These values indicate minimal differences between the groups. The CTQ total score had a Cohen’s d of -0.38, which indicates a small to moderate effect size.

For the categorical variables, we found varying strengths of association: an OR of 1.525 (*p* = 0.495) for sex, 1.857 (*p* = 0.017) for current depressive disorder, 3.045 (*p* < 0.001) for current anxiety disorder, 3.555 (*p* < 0.001) for current PTSD, 1.697 (*p* = 0.203) for current substance use disorder, 1.440 (*p* = 0.187) for personal history of suicide attempts, and 1.401 (*p* = 0.188) for nicotine consumption. While some of these associations were statistically significant and suggested moderate to large effect sizes (current depressive disorder, current anxiety disorder, current PTSD), others indicated only modest effects that are not statistically significant.

### Multivariate analysis

The study investigated whether the link between CTE and OCD is influenced by anxiety symptoms, depressive disorder, PTSD, and eating disorders, focusing on specific trauma subtypes and the overall childhood trauma score.

To evaluate the independent role of each variable, we generated five models (Table 3). Model 2 (sexual abuse) had the lowest AIC (273.84; *p* < 0.001), offering the best fit for the data, followed by Model 3 (physical neglect; AIC = 277.25; *p* < 0.001). The results showed that sexual abuse (Model 2) was significantly associated with OCD in ED patients (*p* = 0.04).

**Table 3:** Multivariate analysis testing if anxiety, depression, PTSD, or ED mediate the CTE-OCD relationship.ª

Independent variables	Model 1	Model 2	Model 3	Model 4	Model 5
Model’s characteristics	AIC = 277.60; *p* < 0.001*	AIC = 273.84; *p* < 0.001*	AIC = 277.25; *p* < 0.001*	AIC = 271.35 *p* < 0.001*	AIC = 273.12 *p* < 0.001*
Current depressive disorder	OR = 0.73; *p* = 0.40	OR = 0.75; *p* = 0.45	OR = 0.77; *p* = 0.48	OR = 1.24; *p* = 0.56	OR = 1.26; *p* = 0.55
Any current anxiety disorder	OR = 2.48; *p* = 0.009*	OR = 2.54; *p* = 0,01*	OR = 2.40; *p* = 0.01*	OR = 0.39; *p* = 0.01*	OR = 0.4; *p* = 0.01*
Current post-traumatic stress disorder	OR = 2.16; *p* = 0.12	OR = 1.56; *p* = 0.40	OR = 2.15; *p* = 0.11	OR = 0.56; *p* = 0.27	OR = 0.65; *p* = 0.42
Eating disorder examination questionnaire (EDE-Q) total score	OR = 2.32; *p* = 0,01*	OR = 2.11; *p* = 0.03*	OR = 2.32; *p* = 0.01*	OR = 2.18; *p* = 0.02*	OR = 2.16; *p* = 0.03*
Childhood trauma subtype	Emotional abuse OR = 1.04; *p* = 0.17	Sexual abuse OR = 1.06; *p* = 0.04*	Physical neglect OR = 1.10; *p* = 0.05	Total CTQ score OR = 1.01; *p* = 0.11	Emotional abuse OR = 1.00; *p* = 0.97
					Sexual abuse OR = 1.05; *p* = 0.16
					Physical neglect OR = 1.06; *p* = 0.32

ªThe first three models refer to different subtypes of CTE: emotional abuse, sexual abuse and physical neglect; the fourth model refers to the total CTQ score, and the fifth model refers to the three subtypes added up

*Asterisks indicate statistical significance (*p*<0.05)

*Abbreviations* PTSD = Post Traumatic Stress Disorder; ED = Eating Disorder; CTE = Childhood Traumatic Experiences; CTQ = Childhood Trauma Questionnaire; OCD = Obsessive Compulsive Disorder; AIC = Akaiae Information Criterion; OR = Odds Ratio

Model 4 (total CTQ score) showed that the severity of the trauma was not significantly associated with OCD in ED patients (*p* = 0.11). Model 5 (sum of the three subtypes of CTE) showed that when combined, neither emotional abuse (*p* = 0.97), sexual abuse (*p* = 0.16), nor physical neglect (*p* = 0.32) were associated with OCD in ED patients.

Additionally, all models (1 through 5) demonstrated that OCD status was significantly associated with the presence of an anxiety disorder at the time of investigation and a more clinically severe ED.

## Discussion

We initially hypothesized that CTE, particularly certain subtypes, could be associated with a risk of comorbid OCD in patients with ED, and that these patients might present with a more clinically severe form of ED more frequently. We found that CTE, most consistently sexual abuse and to a lesser extent physical neglect, had a significant association with OCD in patients with ED.

Furthermore, participants with comorbid ED and OCD tend to have more clinically severe ED and more anxiety disorders compared to those with ED without OCD.

ED and OCD share considerable biogenetic overlap. As previously stated, ED patients are 8.9 times more likely to develop lifetime OCD traits [7] with a lifetime prevalence of OCD ranging from 9.5 to 62% in patients with ED [30]. These observations have been widely reported in the AN subtype, with a firm association between AN and OCD [7]. This includes shared neurobiological gene expression, enriched basal ganglia and medium spiny neurons [31], significant familial aggregation, moderate but significant genetic overlap in twin studies [30], and common prefrontal cortex expression anomalies influencing synaptic conduction [32]. This raises the question of a hypothetical integrated spectrum, as if ED and OCD were different phenotypes of a single disease [33]. The link or coexistence of both disorders has important implications for the therapeutic approach. Indeed, in line with our findings, patients with comorbid ED and OCD have more severe eating difficulties [34]. Implementing tailored OCD treatment in patients with both diagnoses has been linked to a reduction in the severity of both OCD and ED [35]. Conversely, studies have shown that patients who do not recover from ED maintain high obsessive-compulsive scores [36].

Exposure to CTE significantly increases the risk of ED and OCD through analogous neurobiological, genetic, and psychosocial correlates [8, 14, 18]. Epidemiological studies analyzing the mean age at onset of these disorders show that approximately 80% of patients develop OCD before age 18 [37]. The mean age at onset for both AN and BN is around 18 years [38], with ED onset classically described in adolescence [39]. In clinical settings, patients presenting with OCD or ED are more frequently associated with a history of sexual abuse [5, 40]. This subtype of trauma triggers the expression of both conditions during late childhood/adolescence, a timeframe known to be a developmental window of vulnerability [41]. Thus, sexual abuse constitutes a predisposing environmental trigger for the emergence of both psychiatric disorders [42]. Notably, sexual abuse is most strongly linked to the occurrence of bulimia nervosa (OR = 2.73) and binge eating disorders (OR = 2.31) subtypes of ED [8].

From another perspective, yet in accordance with previous epidemiological statements linking ED to OCD, both disorders share similar phenotypes: perfectionism, rigidity, persistency, and excessive ritual behaviors in an attempt to control long-term anxiety and fear [43]. These characteristic phenotypes are best described in the anorexia nervosa subtype [32], and should be taken into account when analyzing the association between sexual abuse, ED, and OCD. However, we should consider that ED patients with OCD may be more likely to recall and report sexual abuse due to their rigid thinking [44]. Moreover, OCD patients may be more prone to disclose sexual abuse events in an attempt to attribute their condition to external factors [45].

Childhood physical neglect increases the risk of both OCD and ED. Among all CTE, physical neglect in OCD patients induces larger right cerebellum volumes due to greater neuronal activation [46, 47], possibly triggering a comorbid dissociative disorder and altering the serotonin transporter gene (5-HTT), thus contributing to treatment resistance [17]. In other words, physical neglect combined with a genetic predisposition increases the likelihood of OCD. Physical neglect is also associated with ED [48]. Self-starvation or binge and purging behaviors in harshly neglected children could be viewed as coping mechanisms to enhance feelings of self-control, in contrast to the abuse context in which they have lost control, yielding self-protective dimensions [4]. Based on a recent meta-analysis, the estimated weighted prevalence of physical neglect in ED patients was 45.4% (95% confidence interval (CI) = 33.1-58.2%) [4], compared to 16.3% in the general population [49], reinforcing the theory of a possible association between a history of physical neglect and an increased risk of developing ED [50]. Our study showed that in ED patients, OCD status was influenced by a history of physical neglect, but when all confounding variables were considered, this association was weaker than the one related to sexual abuse. This suggests that physical neglect is not only directly associated with OCD in patients with ED but might also be indirectly associated through other factors such as a higher propensity for anxiety disorders in this specific population.

Nevertheless, when we combined the three subtypes of CTE, sexual abuse lost its significance. Sexual abuse, when concurrent with physical neglect and emotional abuse, loses its association with OCD in ED patients. We can hypothesize that the subtype of CTE holds significance when it occurs alone but not when associated with other types of trauma, in which case the nature of the trauma loses its significance. The conjunction of multiple traumas could have a broad psychopathological effect, with the emergence of multiple comorbidities that obscure the specificities of a particular subtype of childhood maltreatment.

Our results showed that a more clinically severe ED is more frequently associated with the comorbidity of ED and OCD in patients with a history of CTE. In other words, this suggests that patients with comorbid ED and OCD tend to have a more severe form of ED compared to patients with ED without OCD. Patients with comorbid conditions often have a poorer response to treatment, leading to more severe symptoms [51]. Comorbid OCD and ED result in worse ED treatment outcomes and prognosis [52]. Patients with ED and comorbid OCD usually require more intensive interventions and higher levels of care [7]. It has also been demonstrated that trauma history is associated with more severe eating psychopathology in both subclinical and clinical samples [53]. Given the results of our study, we could speculate that ED patients with a trauma history are more at risk for comorbid OCD and a more severe presentation of their ED.

A number of limitations must be considered when interpreting our data. First, we could not compare the ages of onset of CTE, ED, and OCD, nor establish a temporal relationship between ED and OCD, because of the structure of the questionnaires. It could be useful in the future to examine whether an earlier age at onset and longer duration of ED could affect the development of OCD in subjects affected by CTE. Second, there may be major differences between chronic/recurrent trauma exposure and isolated traumatic events, which could not be captured in our study. Third, we did not compare the different subtypes of ED and disentangle their association with OCD status. This limitation arises from the fact that the methods for evaluating dimensions and subtypes have evolved throughout the study period, which impacted our ability to accurately determine the prevalence of specific eating disorder subtypes. Indeed, current OCD comorbidity seems to be significantly, but inconsistently across studies, higher in AN (14%) than in BN (9%) [8, 52]. Fourth, childhood trauma assessments were made retrospectively using self-report questionnaires, raising the question of reliability and validity of long-term recall despite the fact that recall bias seems to be minimal in CTE [54]. Fifth, the physical neglect subscale demonstrated poor reliability, as responses were inconsistent. Despite this issue, we retained the results for transparency, given that the findings related to physical neglect are not central to the main conclusions of the study. Finally, it was intricate to differentiate between the different subtypes of CTE, hence we considered the CTQ as a continuous variable and evaluated its effect accordingly. A meaningful overlap should have appeared in the analysis model that considers the total CTQ score, but it did not show a significant effect in the multivariate model. Using the lowest AIC helped to indicate which subtype—in case of an overlap—is more associated with the comorbidity of ED and OCD.

This association between OCD, ED, and CTE could pave the way for a new approach to the management of these patients, particularly a transdiagnostic intervention. Research has shown that many disparate psychiatric diagnoses share underlying vulnerabilities, resulting in the development of transdiagnostic interventions designed to treat underlying vulnerabilities and psychopathologic dimensions rather than just one disorder. Transdiagnostic treatments focus on targeting psychological processes or fundamental vulnerabilities known to play a role in the onset and maintenance of classes of disorders [55]. Future research is needed to capture the underlying vulnerabilities between OCD, ED, and CTE, to better elaborate a transdiagnostic intervention that could help treat these patients.

## Conclusions

Findings in this study have shown that CTE, especially sexual abuse, increases the risk of comorbid OCD in ED patients. The association between OCD and ED in patients with a history of CTE correlates with more clinically severe ED and anxiety disorders. Thus, our data indicate the need for focused attention to CTE and OCD screening in ED patients for better prognostic accuracy and tailored treatment.

## Data Availability

No datasets were generated or analysed during the current study.

## Abbreviations

AIC: Akaike Information Criterion
AN: Anorexia Nervosa
BED: Binge-eating disorder
BMI: Body Mass Index
BN: Bulimia nervosa
CI: Confidence interval
CRH: Corticotrophin-releasing hormone
CTE: Childhood traumatic experiences
CTQ: Childhood Trauma Questionnaire
ED: Eating disorders
EDE-Q: Eating Disorder Examination Questionnaire
ITQ: Interquartile
LC-NA: Locus coeruleus-noradrenergic
LHPA: Limbic-hypothalamic-pituitary-adrenal
MINI: Mini International Neuropsychiatric Interview
OCD: Obsessive-compulsive disorder
OR: Odds Ratio
PTSD: Post-traumatic stress disorder
